# Phylogenomics of weevils revisited: data curation and modelling compositional heterogeneity

**DOI:** 10.1098/rsbl.2023.0307

**Published:** 2023-09-20

**Authors:** Yan-Da Li, Michael S. Engel, Erik Tihelka, Chenyang Cai

**Affiliations:** ^1^ State Key Laboratory of Palaeobiology and Stratigraphy, Nanjing Institute of Geology and Palaeontology, Chinese Academy of Sciences, Nanjing 210008, People's Republic of China; ^2^ Bristol Palaeobiology Group, School of Earth Sciences, University of Bristol, Life Sciences Building, Tyndall Avenue, Bristol BS8 1TQ, UK; ^3^ Division of Invertebrate Zoology, American Museum of Natural History, Central Park West at 79th Street, New York, NY 10024-5192, USA

**Keywords:** Curculionoidea, Belidae, phylogeny, compositional heterogeneity

## Abstract

Weevils represent one of the most prolific radiations of beetles and the most diverse group of herbivores on land. The phylogeny of weevils (Curculionoidea) has received extensive attention, and a largely satisfactory framework for their interfamilial relationships has been established. However, a recent phylogenomic study of Curculionoidea based on anchored hybrid enrichment (AHE) data yielded an abnormal placement for the family Belidae (strongly supported as sister to Nemonychidae + Anthribidae). Here we reanalyse the genome-scale AHE data for Curculionoidea using various models of molecular evolution and data filtering methods to mitigate anticipated systematic errors and reduce compositional heterogeneity. When analysed with the infinite mixture model CAT-GTR or using appropriately filtered datasets, Belidae are always recovered as sister to the clade (Attelabidae, (Caridae, (Brentidae, Curculionidae))), which is congruent with studies based on morphology and other sources of molecular data. Although the relationships of the ‘higher Curculionidae’ remain challenging to resolve, we provide a consistent and robust backbone phylogeny of weevils. Our extensive analyses emphasize the significance of data curation and modelling across-site compositional heterogeneity in phylogenomic studies.

## 1. Introduction

Weevils (superfamily Curculionoidea) represent a hyperdiverse and globally distributed group of phytophagous beetles, with approximately 62 000 described species in 5800 genera [1,2]. Most species of Curculionoidea can be easily recognized by their elongate rostrum, which has been suggested as a key innovation responsible for their success [3–6]. Indeed, the rostrum permitted weevils to access a greater variety of plant tissues than their relatives, particularly when coupled with the later appearance of an elongate scape (mostly in Curculionidae) allowing the antenna to fold posteriorly and for the rostrum to extend deeper into plant tissues while simultaneously preserving some motility from the pedicel to apex. These two characters together allowed weevils to further extend their feeding habits as well as to coopt the rostrum for chewing deep oviposition sites, and with this the higher weevils proliferated in species and dietary breadth. Although there are still some different opinions (e.g. [6–8]), most recent classifications have reached a consensus on the familial division of Curculionoidea (see [1,2]). Clarke *et al*. [9] recognized eight extant curculionoid families, i.e. Cimberididae, Nemonychidae, Anthribidae, Belidae, Attelabidae, Caridae, Brentidae and Curculionidae, plus one extinct family, Mesophyletidae, known exclusively from the mid-Cretaceous Burmese amber. The more plesiomorphic Nemonychidae, Belidae and Caridae are commonly associated with gymnosperms, whereas the most species-rich Brentidae and Curculionidae are predominantly associated with angiosperms [2]. This niche shift is apparently associated with the radiation of angiosperms, which probably contributed to the success of weevils (e.g. [10–12]).

The phylogeny of Curculionoidea has been investigated extensively based on both morphological and molecular data (e.g. [13–21]). These studies have generally established a congruent framework for the interfamilial relationships of curculionoids. However, in a recent anchored hybrid enrichment (AHE)-based study by Shin *et al*. [21], Belidae were strongly recovered as the sister group of the clade encompassing Nemonychidae + Anthribidae when analysed using amino acid (AA) sequences (their preferred topology). This position of Belidae in Shin *et al*. [21] was unexpected and contradicted previous studies based on either morphological or molecular data sets. In particular, in the most comprehensive phylogenomic studies of beetles based on dozens of nuclear protein-coding genes [22,23] or genome-scale data [12], Belidae were consistently and strongly resolved as sister to the group composed of Attelabidae, Caridae, Brentidae and Curculionidae.

Anchored hybrid enrichment data [24,25], typically represented by hundreds of loci from across the genome, have provided invaluable and novel insights into both deeper and shallower phylogenies of vertebrates [26–29], invertebrates [30–33] and plants [34,35]. AHE-based phylogenomics often yield a congruent tree with the topology inferred from other large-scale data sets (e.g. transcriptomes and ultraconserved elements), when the process of molecular evolution is more adequately modelled [36]. As such, the abnormal phylogenetic position of Belidae recovered in Shin *et al*. [21] could have resulted from model misspecification and/or data curation, since both aspects have been shown as crucial for tree inference in the phylogenomic era [37–40]. In terms of modelling molecular evolution of large-scale data sets, the site-heterogeneous models such as CAT-GTR, provide a consistently better fit to the data and outperform the site-homogeneous substitution models, at the expense of a higher computational cost.

In the present study, we reanalyse the data by Shin *et al*. [21] using various models of molecular evolution and data filtering methods, aiming to build a backbone phylogeny of weevils and clarify the phylogenetic position of Belidae.

## 2. Material and methods

### (a) Data preparation

Our analyses were conducted on four datasets prepared based on the concatenated AA sequences by Shin *et al*. [21]. We did not use the nucleotide (NT) data set because of the presence of compositional biases and apparently saturated third-codon positions [21]. More importantly, it has long been shown that AA-based phylogenetic results are more robust for resolving deep divergences (e.g. [23,41]). Dataset 1 was the same as the original matrix by Shin *et al*. [21]. Datasets 2–4 were trimmed with different methods to remove ambiguously aligned regions and reduce compositional heterogeneity. Dataset 2 was trimmed with BMGE 1.1 [42] using BLOSUM95 similarity matrix and the default entropy score cut-off (-h 0.5). An inspection revealed some misaligned regions unremoved in the BMGE-trimmed dataset 2. Figure S1 (see electronic supplementary material) provides an example of these misaligned regions, where two obviously discrete sequence modes can be seen: the predominant one starting with FEEVQL, and the less common one starting with XEGYPV. The XEGYPV-type sequences are present in unrelated lineages. According to NCBI BLAST searches, these two types of sequences are different parts of the same RNA helicase, which are clearly non-orthologous. Likewise, most other misaligned regions are also represented by different parts of the same protein. Therefore, for dataset 3, after trimming by BMGE, these incorrectly aligned regions were further manually removed. Dataset 4 was trimmed with Gblocks 0.91 [43] under a relaxed setting (minimum number of sequences for a flank position: minimum; maximum number of contiguous non-conserved positions: maximum; minimum length of a block: minimum; allowed gap positions: with half), as the default settings have been suggested to be too stringent [42]. The distant outgroup *Aethina* (Nitiduloidea: Nitidulidae) was removed in datasets 2 and 3.

### (b) Testing compositional heterogeneity

To assess the compositional heterogeneity across taxa, the normalized relative composition frequency variability (nRCFV) was calculated using RCFV Reader v.1 [44]. To assess the compositional heterogeneity across sites, we tested the model fitness (based on the Bayesian information criterion, BIC) of various site-homogeneous models alongside the mixture models LG4X [45] and LG + C20 + F [46] using IQ-TREE 1.6.2 or 2.1.3 [47,48]. The best fitting site-homogeneous model was determined with ModelFinder implemented in IQ-TREE 2.1.3 [49], where several new clade-specific AA replacement matrices were available [50].

### (c) Phylogenetic analyses

The maximum likelihood (ML) phylogenetic trees were inferred with IQ-TREE 1.6.2 or 2.1.3 under both the best fitting site-homogeneous model and the mixture models LG4X, LG + C20 + F, and LG + C40 + F (for dataset 2 only), as well as the posterior mean site frequency (PMSF) approximation of LG + C20 + F, LG + C40 + F and LG + C60 + F [51]. The PMSF profiles were computed based on the guide tree estimated under LG4X (datasets 1, 2, 4) or LG + C20 + F (dataset 3). For each ML analysis, ultrafast bootstrap support values were calculated from 1000 replicates [52]. The gene concordance factor (gCF) and site concordance factor (sCF) were calculated for the LG4X tree based on dataset 1 [53].

The Bayesian phylogenies were inferred under the infinite mixture model CAT-GTR + G4 [54] in PhyloBayes MPI 1.7 [55]. Two independent chains were computed for each Bayesian analysis, with their convergence evaluated using the bpcomp program. Ideally the analyses were stopped when the maximum difference across bipartitions (maxdiff) was at least lower than 0.3 (but see also Results). The trees were visualized using the online tool iTOL 6.8 [56].

The coalescent-based phylogenies were also estimated. Since the sequences of individual genes were not directly supplied by Shin *et al*. [21], we extracted the aligned protein-coding genes (in AA form) from the supermatrix based on the partition file provided, using R 4.1.0 [57] and R packages Biostrings 2.62.0 [58] and seqinr 4.2-30 [59]. The gene trees were estimated under the LG4X model using IQ-TREE 2.1.3. The species tree was then inferred using ASTRAL 5.7.8 [60], first with all 521 gene trees as the input. As some of the gene fragments contain clearly misaligned regions (as discussed above), an additional species tree was estimated based on 364 gene trees (157 trees based on misaligned fragments were excluded). The species trees were visualized using R package ggtree 3.2.1 [61], with the quartet supports mapped using R package ggimage 0.3.3 [62].

## 3. Results

### (a) Compositional heterogeneity in datasets

The normalized RCFV value measures the compositional heterogeneity across taxa. The original dataset displayed a relatively high nRCFV value, indicating a pronounced compositional heterogeneity (table 1). In the trimmed datasets, the across-taxa compositional heterogeneity was reduced to some extent and consequently, the data occupancy was increased significantly from 22.1% (dataset 1, the original AA dataset) to 86.4% (dataset 2), 86.6% (dataset 3) and 78.4% (dataset 4) (table 1). As shown by the BIC values (table 1), for each of the datasets, the best fitting site-homogeneous model outperformed the LG4X model. However, overall the mixture model LG + C20 + F (and also LG + C40 + F when computed) always had better fitness than the best site-homogeneous model selected by ModelFinder implemented in IQ-TREE. This implies a high level of compositional heterogeneity across sites, and shows the necessity of analysing with the site-heterogeneous model.

**Table 1.** Comparison among the differently filtered datasets. BIC, Bayesian information criterion; nRCFV, normalized relative composition frequency variability.

		dataset 1	dataset 2	dataset 3	dataset 4
number of amino acid sites		225 254	25 468	23 329	39 572
data occupancy		22.1%	86.4%	86.6%	78.4%
nRCFV		0.003760	0.000979	0.000935	0.001243
BIC score	LG + R6	3 661 622.351	774 637.579	625 241.560	1 335 347.454
	Q.insect + R6	3 645 847.536	769 033.791	620 442.561	1 327 483.076
	LG4X	3 674 201.090	777 927.453	629 723.247	1 339 488.312
	LG + C20 + F	3 593 307.319	750 270.935	610 507.371	1 294 237.132
	LG + C40 + F	/	747 484.080	/	/

### (b) Phylogeny of Curculionoidea

The Bayesian analyses under the CAT-GTR model did not fully converge for datasets 1 and 4 (maxdiff = 1). However, the failure to converge was due only to discrepancies between chains about the nodes within the family Curculionidae, and all the nodes related to the interfamilial relationships of Curculionoidea had reached a posterior probability support of 1.00 in these two analyses (electronic supplementary material, figures S8 and S30). According to [63], these results should be acceptable as the discrepancies do not directly affect our nodes of interest.

**Figure 1.**
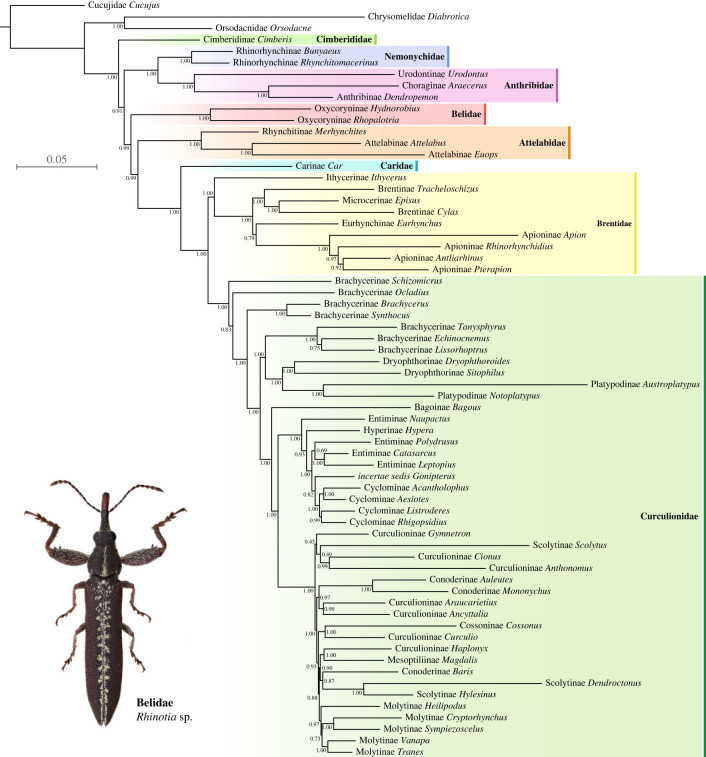
Bayesian phylogeny of Curculionoidea, analysed based on the AHE amino acid dataset 3 (trimmed with BMGE and manually) under the CAT-GTR + G4 model in PhyloBayes. The support values represent Bayesian posterior probabilities.

**Figure 2.**
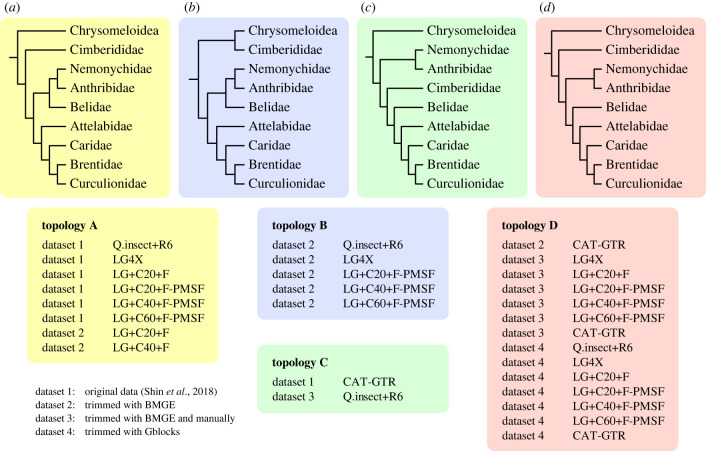
Comparison among tree topologies generated with different models and datasets. Topology D is our preferred topology for weevil phylogeny.

The relationships among most families were stable across our analyses with different datasets and models, except for the positions of Cimberididae and Belidae (figures 1 and 2). A backbone of ((Nemonychidae, Anthribidae), (Attelabidae, (Caridae, (Brentidae, Curculionidae)))) was consistently recovered, which was accordant with the original results by Shin *et al*. [21]. In most analyses, Cimberididae were resolved as sister to the remaining Curculionoidea (figure 2). In a few analyses, Cimberididae appeared as sister to the chrysomeloid outgroups Orsodacnidae + Chrysomelidae, sister to the remaining Curculionoidea except Nemonychidae + Anthribidae (figure 2), or sister to Nemonychidae + Anthribidae (electronic supplementary material, figure S33), which we suppose was an artefact. The internal relationships of Curculionidae were somewhat variable among analyses and not well supported, suggesting that the present data are insufficient to resolve these issues.

### (c) Position of Belidae

Belidae were recovered as sister to Nemonychidae + Anthribidae in most of the results from datasets 1 and 2, except when analysed with the infinite mixture model CAT-GTR (figure 2). However, under the CAT-GTR model, Belidae turned out to be sister to the ‘ACBC clade’ of (Attelabidae, (Caridae, (Brentidae, Curculionidae))) (figure 2). In all the results based on datasets 3 and 4, even including those based on site-homogeneous models, Belidae were also recovered as sister to the ACBC clade (figure 2). In addition, at least in the results under the CAT-GTR model, the placement of Belidae was always strongly supported (BPP = 0.99–1.00). Our coalescent analyses supported Belidae as sister to the ACBC clade as well, although the quartet support was not significantly higher than the alternative hypotheses (electronic supplementary material, figures S33, S34) [64].

## 4. Discussion

In the partitioned maximum-likelihood analysis of AA data by Shin *et al*. [21], Belidae were grouped together with Nemonychidae and Anthribidae. This topology conflicted with the NT-based results by themselves and also previous studies [16,20] (see also [65]), where Belidae were often grouped with the remaining Curculionoidea except Nemonychidae and Anthribidae. Shin *et al*. [21] suggested that the position of Belidae in the NT-based results might be an artefact resulting from codon-usage bias. However, the overall low concordance factors of the ML tree suggest that the phylogenetic signal is quite weak (electronic supplementary material, figures S31, S32), and particularly, the sCF supporting Belidae as sister to Nemonychidae + Anthribidae is even lower than the discordance factor for an alternative quartet. In this case, the removal of phylogenetic noise and the use of appropriate evolutionary models could be of vital importance. Our re-analysis of the AA dataset by Shin *et al*. [21] shows that Belidae are grouped together with the clade (Attelabidae, (Caridae, (Brentidae, Curculionidae))), as long as the molecular data are properly filtered and (or) the infinite mixture model is adopted. This topology is in accordance with recent large-scale phylogenomic studies [12,22,23]. It is noteworthy that the coalescent tree based on AA data revealed the same interfamilial relationship (electronic supplementary material, figure S34; fig. S14 in [21]) as our preferred tree (figure 1). Actually, as shown by previous studies, methods using coalescent or site-heterogeneous models can often reconstruct more plausible phylogenies, as they better reflect the real evolutionary process (e.g. [66–68]).

In the original study by Shin *et al*. [21], the site-heterogeneous CAT-GTR model was also applied to both AA and NT datasets (figs. S12, S13 in [21]). They ran two independent chains using PhyloBayes MPI 1.7 [55] for each dataset and retrieved maxdiff values of 1, due to the size of the data and conflicts within the CEGH (Cyclominae, Entiminae, Gonipterini and Hyperinae) and CCCMS (Conoderinae, Cossoninae, Curculioninae, Molytinae and Scolytinae) clades. This MCMC mixing problem is not unexpected for large datasets with more than 20 000 positions, as it was also shown in our reanalysis of their original AA dataset. Surprisingly, in the posterior consensus trees derived from their PhyloBayes analyses, all nodes of their AA and NT trees were maximally supported (BPP = 1). Such results are suspect, because a maxdiff of 1 means that at least one clade is inferred with a posterior probability of 1 in one chain and 0 in the other chain and therefore all BPPs of 1 in the consensus trees of the two chains are impossible by definition. It therefore seems possible that their maximally supported trees were derived from only one chain of each PhyloBayes analysis, but this is an inappropriate practice. Contrary to Shin *et al*. [21], in our CAT-GTR analysis of the same original AA dataset, Belidae were strongly supported as the sister group of the clade (Attelabidae, (Caridae, (Brentidae, Curculionidae).

The genomic data used by Shin *et al*. [21] were obtained through anchored hybrid enrichment. While efficient for collecting a large amount of data, such target sequence capture approaches could introduce considerable missing data [24], which might affect the accuracy of alignment and phylogenetic reconstruction [69]. As shown in table 1, the original concatenated datasets were very gappy and the data occupancy was low (22.1%). This is likely because they retained the flanking regions of a distant outgroup (*Aethina*), included some incorrectly aligned loci, and skipped the data filtering step before tree reconstruction. The impact of alignment trimming on downstream phylogenetic analysis is often not very clear [70], as it is challenging to achieve balance between removing potentially misleading alignment regions and removing phylogenetically informative regions [71,72]. Nevertheless, in our analyses based on the trimmed datasets 3 and 4, Belidae are always recovered in a stable position regardless of model choice and were congruent with analyses using the heterogeneous CAT-GTR model, indicating that proper trimming or data curation is crucial at least for the present dataset.

The systematic placement of Belidae based on our reanalyses is congruent with morphology-based studies [13–15] (see also [17,73]). Potential synapomorphies for the clade including Belidae, Attelabidae, Caridae, Brentidae and Curculionidae include: hypopharyngeal bracon without sclerome, frontoclypeal suture distinct, mandibular molar absent and maxillary articulatory lobes absent in larvae; and clypeolabral suture indistinct, mandibular pharyngeal process at least as long as mandible, mandibular molar absent, mandible with teeth at incisor area, maxillary palp short and spermathecal duct and gland contiguous or subcontiguous on spermathecal body in adults [13–15]. Actually, it is not uncommon for a counterintuitive (i.e. contradictory to morphology) phylogeny based on simple models to be later proved as an artefact by analyses with the better-fitting site-heterogeneous models (e.g. [37,74,75]).

Although the interfamilial relationships of weevils (Curculionoidea) are now confidently resolved, the relationships within the ‘higher Curculionidae’ [21], especially the CCCMS clade, remain unsettled due to ancient rapid radiation likely during the Late Cretaceous as indicated by exceptionally short internodes. A more focused phylogenomic study of Curculionidae based on genome-scale data (e.g. UCEs, nuclear genomes) would be welcome to unravel the mysterious rapid radiation of derived weevils.

## 5. Concluding remarks

Weevils represent a classical case study on adaptive radiation associated with the diversification of flowering plants [10,11]. A valid phylogeny would be crucial to reconstructing the sequence of character acquisitions that enabled weevils to attain their present-day mega-diversity, and the timescale of their radiation. Here we provide a well-resolved backbone phylogeny of weevils, clarifying the interfamilial relationships of extant weevils. As recovered in other recent phylogenomic studies [12,23], Belidae are confirmed as the sister group of (Attelabidae, (Caridae, (Brentidae, Curculionidae))) based on the re-analysis of the AHE AA data by Shin *et al*. [21]. This topology is well accordant with previous studies based on morphology and other sources of molecular data. Our results further demonstrate the importance of data curation and modelling compositional heterogeneity in genomic-scale phylogenetic analyses of insects.

## Data Availability

The data are provided in the electronic supplementary material [76].
